# Hsa_circ_0087302, a circular RNA, affects the progression of osteosarcoma via the Wnt/β-catenin signaling pathway

**DOI:** 10.7150/ijms.69501

**Published:** 2022-08-01

**Authors:** Lijiao Peng, Qianzheng Liu, Tingrui Wu, Peng Li, Yixia Cai, Xinjian Wei, Yuming Zeng, Junhong Ye, Peicong Chen, Jing Huang, Hao Lin

**Affiliations:** 1 Department of Orthopedics, Affiliated Hospital of Guangdong Medical University, Zhanjiang, Guangdong, 524001, China.; 2 Oncology Hospital, Affiliated Hospital of Guangdong Medical University, Zhanjiang, Guangdong, 524001, China.; 3 Stem Cell Research and Cellular Therapy Center, Affifiliated Hospital of Guangdong Medical University, Zhanjiang, Guangdong, 524001, China.

**Keywords:** Hsa_circ_0087302, Osteosarcoma, Oncogenic circRNA, Wnt/β-catenin

## Abstract

Osteosarcoma is the most common malignant tumor in adolescent bone malignancies. It has the characteristics of a high metastasis rate, high mortality and poor prognosis. As a subclass of endogenous noncoding RNAs, circRNAs have been identified to be related to the occurrence, development and prognosis of different kinds of cancers, but the mechanism of their effect on osteosarcoma is not clear. In the present study, we identified a novel circRNA, hsa_circ_0087302, by RNA-seq, and we found that it was expressed at low levels in osteosarcoma. Using RT-PCR, we confirmed that the expression of hsa_circ_0087302 in osteosarcoma cells was lower than that in osteoblasts. Functional validation experiments revealed that hsa_circ_0087302 overexpression inhibited proliferation, cell cycle, migration, and invasion in osteosarcoma cells. Furthermore, Western blotting experiments demonstrated that hsa_circ_0087302 affected the expression of cell cycle- and Wnt/β-catenin signaling pathway-related proteins. For the first time, we identified that hsa_circ_0087302 may affect the malignant biological behavior of osteosarcoma cells through the Wnt/β-catenin signaling pathway. In summary, hsa_circ_0087302 may provide a new direction for the diagnosis and treatment of osteosarcoma.

## Introduction

Osteosarcoma is the most common malignant bone tumor in adolescents 1. According to statistics, the incidence rate of osteosarcoma is three parts per million in China, accounting for 5% 2 of young people's malignant tumors. The incidence rate of osteosarcoma is increasing year by year and is the main cause of cancer death among adolescents. Because of the lack of specific diagnostic markers and detection indicators, approximately 20% of patients have imperceptible micrometastasis at the first diagnosis 3,4. In recent decades, a comprehensive treatment strategy based on surgery combined with chemotherapy has become the standard treatment for osteosarcoma, and the 5-year survival rate has increased from 70% to 82% 5. However, surgical resection often affects the motor function of patients and has a high disability rate. Mainstream chemotherapy drugs have large side effects and lack of specificity, resulting in poor treatment effect and prognosis. The high degree of malignancy, metastasis in the early stage and recurrence after treatment are important factors affecting the overall survival of osteosarcoma. Experiments have demonstrated that the development of osteosarcoma is closely related to risk factors, such as age, sex, height and family inheritance 6-8; however, the exact etiology of osteosarcoma is not clear, and the pathogenesis of osteosarcoma needs to be further clarified. Therefore, for the diagnosis and therapy of osteosarcoma, it is essential to study the molecules related to the pathogenesis of osteosarcoma, identify specific biomarkers and screen clinical early warning and treatment targets.

As an emerging, widespread and evolutionarily conserved non-coding RNA, circular RNA (circRNA) has become a hotspot in life science research in recent years. In 1976, the first circular RNA was found in plant-infected viroid and parainfluenza virus particles 9, and it was later detected in humans and animals 10. According to the difference of origin, circular RNAs are divided into the following three categories: exon circRNAs (ecRNAs) 11,12, exon-intron circRNAs (elciRNAs) 13 and circular intron RNAs (circRNAs) 14. Existing studies have shown that circRNA has various biological functions, including acting as a miRNA sponge, acting as a protein sponge, interacting with RNA-binding proteins, selective splicing, transcriptional regulation, encoding proteins and encoding tumor-related functional peptides 15.Among them, the function of circRNA-encoded proteins on the occurrence and development of diseases is controversial, and studies have found emerging functions of circRNA-encoded proteins in human cancers 16. Circular RNA may be related to the occurrence and progression of many diseases, and it is expected to be a new biomarker of diseases. Recently, a growing amount of evidence has shown that circRNAs play an essential role in the occurrence and development of tumors. Circular RNAs are related to a variety of cancers and diseases, such as laryngeal cancer, colon cancer, lung cancer, liver cancer, renal cancer, cervical cancer, gastric cancer, esophageal cancer, bladder cancer, breast cancer, glioma, leukemia and lymphoma 17-29. Although an increasing number of circular RNAs have been identified, little is known about the specific mechanism of circular RNA in osteosarcoma. In the present study, we identified a novel circular RNA, namely hsa_circ_0087302, from RNA sequencing data, and we found that it was downregulated in osteosarcoma cells by RT-PCR. Overexpression of hsa_circ_0087302 inhibited cell proliferation, migration and invasion. Through the enrichment analysis of KEGG pathways, we found that hsa_circ_0087302 was closely related to lysine degradation, bacterial invasion of epithelial cells, the ErbB signaling pathway, protein processing in the endoplasmic reticulum, transcriptional misregulation in the cancer pathway and central carbon metabolism in cancer. Cell cycle experiments and Western blot analysis showed that most osteosarcoma cells overexpressing hsa_circ_0087302 were arrested in G0/G1 phase, and hsa_circ_0087302 overexpression affected the expression of cyclin D1, a cycle-associated protein. Because cyclin D1 is the downstream target gene of the Wnt/β-catenin signaling pathway and the Wnt/β-catenin signaling pathway has been confirmed to be involved in the malignant biological behavior of osteosarcoma, we detected the key proteins in the Wnt/β-catenin signaling pathway by Western blot analysis, and we found that overexpression of hsa_circ_0087302 affected their expression level. Thus, hsa_circ_0087302 may affect the biological behavior of osteosarcoma through the Wnt/β-catenin signaling pathway.

## Materials and methods

### Cell Lines and Culture

Human osteoblasts (hFOB1.19) and human osteosarcoma cells (HOS, MG63, U2OS and 143B) were purchased from the Chinese Academy of Sciences Cell Bank. hFOB1.19 cells were cultured in D-MEM/F-12 medium (Gibco, Carlsbad, CA, USA) containing 10% fetal bovine serum (FBS) (Gibco, Sydney, Australia) at 33.5 °C and 5% CO_2_. HOS and MG63 cells were cultured in MEM (Gibco, Carlsbad, CA, USA), and U2OS cells were cultured in RPMI-1640 medium (Gibco, Carlsbad, CA, USA). In addition, 143B cells were grown in D-MEM/F-12 medium. All human osteosarcoma cells were cultured in medium containing 10% FBS at 5% CO2 and 37 °C. Since human osteoblasts (hFOB1.19) and human osteosarcoma cells (HOS, MG63, U2OS and 143B) were grew adherently, the cells were digested with trypsin containing 0.25% Trypsin-EDTA.

### RNA sequencing assay

Total RNA was isolated from cells according to the instructions of the HiPure Total RNA Mini Kit (Magen). The integrity of total RNA was determined by an Agilent 2100 Bioanalyzer (Applied Biosystems, Carlsbad, CA, USA), and the concentration of total RNA was detected by a Qubit 3.0 Fluorometer (Invitrogen, Carlsbad, CA, USA).

The KAPA RNA HyperPrep Kit with RiboErase (HMR) for Illumina® (Kapa Biosystems, Inc., Woburn, MA) was used to remove rRNA, and fragmentation buffer was added to the obtained mRNA to make short fragments. The fragmented mRNA was then used as a template, and six-base random primers (random hexamers) were used to synthesize the first strand of cDNA. To synthesize the second strand of cDNA, buffer, dNTPs, RNase H and DNA polymerase I was added, and it was purified by QiaQuick PCR kit and eluted with EB buffer. End repair was performed, and an adenine base was added. Sequencing adapters were added, and the target size fragments were recovered by agarose gel electrophoresis. A qPCR-based KAPA Biosystems Library Quantification kit (Kapa Biosystems, Inc., Woburn, MA, USA) was used to determine the accurate quantification of the sequencing application. The library preparation work was then completed, and the constructed library was sequenced.

### Functional enrichment analysis

The KEGG pathway enrichment site (http://www.genome.jp/KEGG/) was used to identify the pathway and functional enrichment of the target gene as well as to draw a pathway map.

### RNA extraction and quantitative real-time polymerase reaction (qRT-PCR)

Total RNA was extracted from cells by using TRIzol reagent (Invitrogen, Carlsbad, CA, USA) according to the manufacturer's instructions, and NanoDrop equipment (Thermo Fisher Scientific, Waltham, MA, USA) was used to quantitatively analyze the extracted total RNA. First-strand cDNA was generated using a cDNA synthesis kit (Takara, Otsu, Japan). TB Green PCR Master Mix (Takara) and ABI StepOne Real-Time PCR System (Applied Biosystems 7500, Foster City, CA, USA) were used for real-time PCR analysis, and the results were analyzed by comparing Ct (2 -ΔΔCt) values. The primer sequences were as follows: hsa_circ_0087302 (forward, 5′-GCTGCTGCTGCTGCCTATGG-3′ and reverse, 5′-CTGGATGCCTGTCAGGTTTGGAG-3′); and GAPDH (forward, 5′-CACCCACTCCTCCACCTTTG-3′ and reverse, 5′-CCACCACCCTGTTGCTGTAG-3′).

### Construction of overexpression vector and stable transfection of cells

Human lentivirus-circ-0087302 and Human lentivirus-NC was purchased from GeneChem (Shanghai, China). Lentiviral vector containing hsa_circ_0087302 was used to construct hsa_circ_0087302 overexpression model (OE-circ-0087302), Lentiviral vector carrying nonsense sequence as negative control (NC). Two lentivirus suspension was added to the six-well plate respectively, which was at a 50% cell density, at a MOI of 50 and cultured for three days. Puromycin (Gibco, Grand Island, NY, USA) was used to screen the successfully transfected osteosarcoma cells for subculture.

### Cell Counting Kit-8 (CCK-8) assay

Approximately 2×10^3^ stably transfected osteosarcoma cells were seeded into 96-well plates and allowed to adhere. At five time points (0, 24, 48, 72 and 96 h), 10 µl of CCK-8 solution was added to each well and incubated at 37 °C for two hours. The optical density (OD) value of the sample was measured at 450 nm using a spectrophotometer.

### Colony Formation Assay

Stably transfected OS cells (500/well) were inoculated into each well of a 6-well plate and incubated in a 37 °C incubator. After MG63 cells were incubated for 5 days and U2OS cells were incubated for 7 days, cells were fixed with 20% methanol for 20 minutes and stained with 0.1% crystal violet for 30 minutes. Colonies with more than 50 cells were counted.

### Cell migration and invasion assays

Stably transfected osteosarcoma cells in logarithmic growth were collected. For the migration assays, 100 µl of cell suspension (2×104 cells) in culture medium without FBS was inoculated into the upper chamber (Costar Inc., USA). For the invasion assays, 100 μl of Matrigel (Corning) was added at a concentration of 250 μg/ml to the upper chamber followed by incubation at 37 °C for 1 hour, and the cell suspension (4.0×104 cells) in culture medium without FBS was then added to the upper chamber. To induce cell migration, 500 μl of medium containing 20% FBS was added to the lower chamber. After MG63 and U2OS cells were incubated for 24 hours in the Migration assays and 36 hours in the invasion assays, cells in the upper chamber were wiped with cotton swabs, fixed with methanol for 20 minutes and stained with crystal violet for 30 minutes. Cells were observed and imaged using an inverted microscope (Olympus, Tokyo, Japan).

### Cell cycle assay

Stably transfected osteosarcoma cells were cultured in six-well plates in a 37 °C incubator for 48 h. Cells were washed three times with PBS after collection and then incubated in 70% ethanol at 4 °C overnight. After washing the cells with PBS, the cells were centrifuged to obtain pellets. 25 μL of propidium iodide (C1052-2; Beyotime, Shanghai, China) and 10 μL of RNase (C1052-3; Beyotime, China) were added to each sample for staining. After incubation at 37 °C for 30 minutes in the dark, the cell cycle was detected by BD FACSCalibur flow cytometer (FACSCalibur, BD, San Jose, CA, USA).

### Western blotting

Stably transfected OS cells were incubated in six-well plates for 48 hours and lysed using RIPA buffer (Solarbio, Beijing, China). Determination of protein concentration using a bicinchoninic acid (BCA) protein assay kit (Beyotime). Then samples were subjected to SDS-PAGE (Solarbio, Beijing, China) at 80 V for 180 minutes and subsequently transferred to a PVDF membrane at 250 mA in transfer solution for 150 minutes. The membrane was blocked with 5% skim milk at room temperature for 1 h and then washed for 30 minutes. The membrane was then incubated with primary antibody at 4 °C overnight and then washed for 30 minutes. The membrane was then incubated with the secondary antibody at room temperature for 1 h. After the membrane was washed, a chemiluminescence and fluorescence imaging system (Tanon 5200, Shanghai, China) was used to detect immune response bands. Rabbit monoclonal antibodies against GAPDH [Cell Signaling Technology (CST), Inc., Danvers, MA, USA] were diluted at 1:1000. Mouse monoclonal antibodies against β-catenin, cyclin D1, Dvl 2, Dvl 3, CD44, LRP 6, LEF 1, Axin, c-Myc and c-Jun [Cell Signaling Technology (CST), Inc., Danvers, MA, USA] were diluted at 1:1000. The secondary antibodies (anti-mouse IgG/HRP) were diluted at 1:5000 [Cell Signaling Technology (CST), Inc., Danvers, MA, USA]. The protein levels were normalized by GAPDH for each sample.

### Statistical analysis

GraphPad Prism 7 was used for all statistical analyses. The measurement data were evaluated by unpaired Student's *t*-test and are expressed as the mean ± standard deviation (SD). A *P* value < 0.05 was considered statistically significant.

## Results

### Expression of circular RNA in osteosarcoma

**Figure 1:**
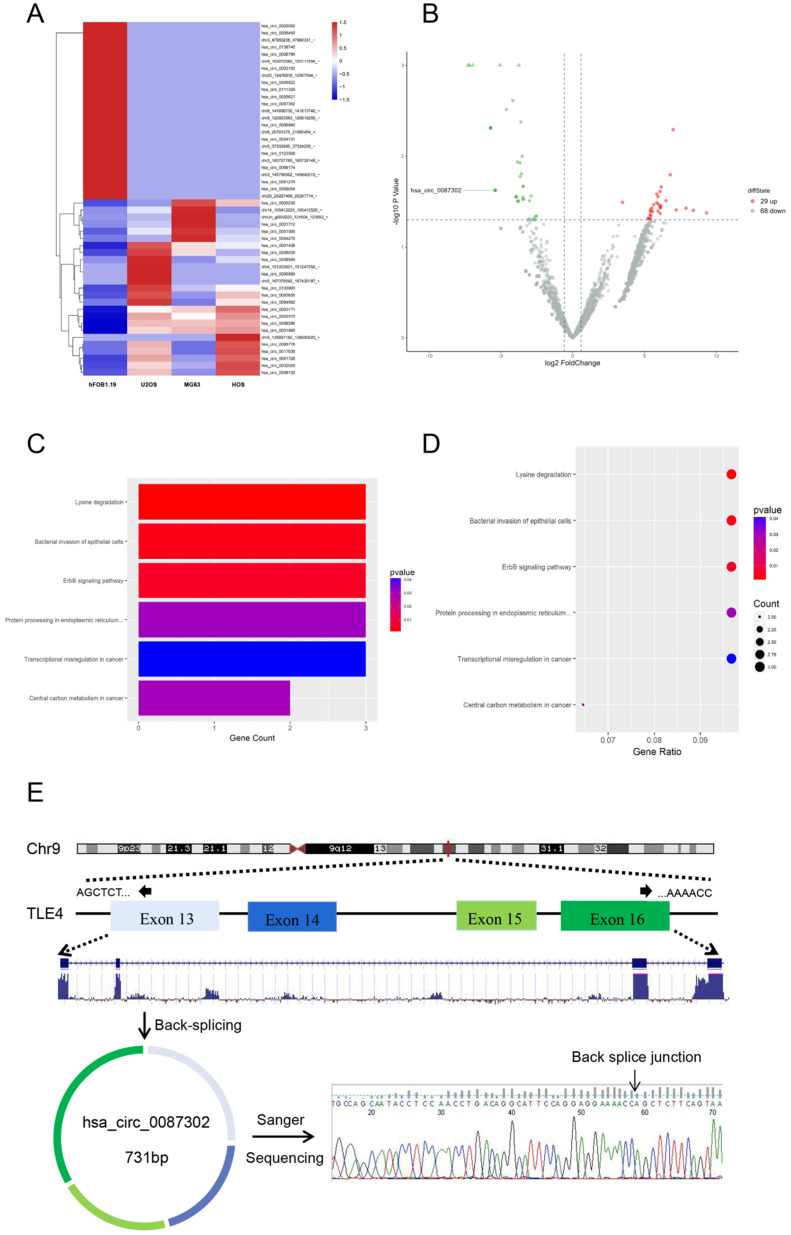
** Differentially expressed circRNAs in osteosarcoma cells and osteoblasts as well as enrichment analysis of related pathways. (A)** Hierarchical cluster analysis of differentially expressed circRNAs. In the heatmap, red represents high expression, and green represents low expression. The columns represent cells, and the rows represent different circRNAs. **(B)** Volcano map showing differentially expressed circRNAs. The vertical line represents a multiple of 1.5, and the horizontal line indicates a p value equal to 0.05. **(C-D)** The KEGG database was used for pathway enrichment analysis of circRNAs. **(E)** Schematic illustration of hsa_circ_0087302 formation and the results of Sanger sequencing.

In order to study the circRNA expression in osteosarcoma cells, we compared the circRNA expression of osteosarcoma cell lines (HOS, MG63 and U2OS) and normal human osteoblast cell lines (hFOB1.19) by RNA-sequencing analysis. After data screening (fold change ≥ 1.5, P value < 0.05, and FDR < 0.05), there were 29 upregulated circRNAs and 68 downregulated circRNAs in osteosarcoma cells (Figure 1B). A hierarchical cluster diagram was generated for the differentially expressed circRNAs (Figure 1A). KEGG analysis showed that the differentially expressed circRNAs were related to six different signaling pathways, including Lysine degradation, Bacterial inwasion of epithelial cells, ErbB signaling pathway, Protein processing in endoplasmic reticulum, Transcriptional misregulation in cancer and Central carbon metabolism in cancer. (Figure 1C-D). By combining the list of downregulated expression circRNAs in the high-throughput sequencing results with the analysis results of circRNA microarray probe data in circbase database (http://www.circbase.org/), we found that hsa_circ_0087302 is formed from the head and tail of exon 13-16 of its parent gene TLE4, which is located on chromosome 9 (chr9: 82319697-82324614), with a length of 731 BP. The presence and structure of hsa_circ_0087302 was confirmed by primer-specific amplification followed by Sanger sequencing of the PCR product. Therefore, we select hsa_circ_0087302 as the research object of this experiment (Figure 1E).

### Hsa_circ_0087302 expression is decreased in osteosarcoma cells, and construction of the hsa_circ_0087302 overexpression model

**Figure 2:**
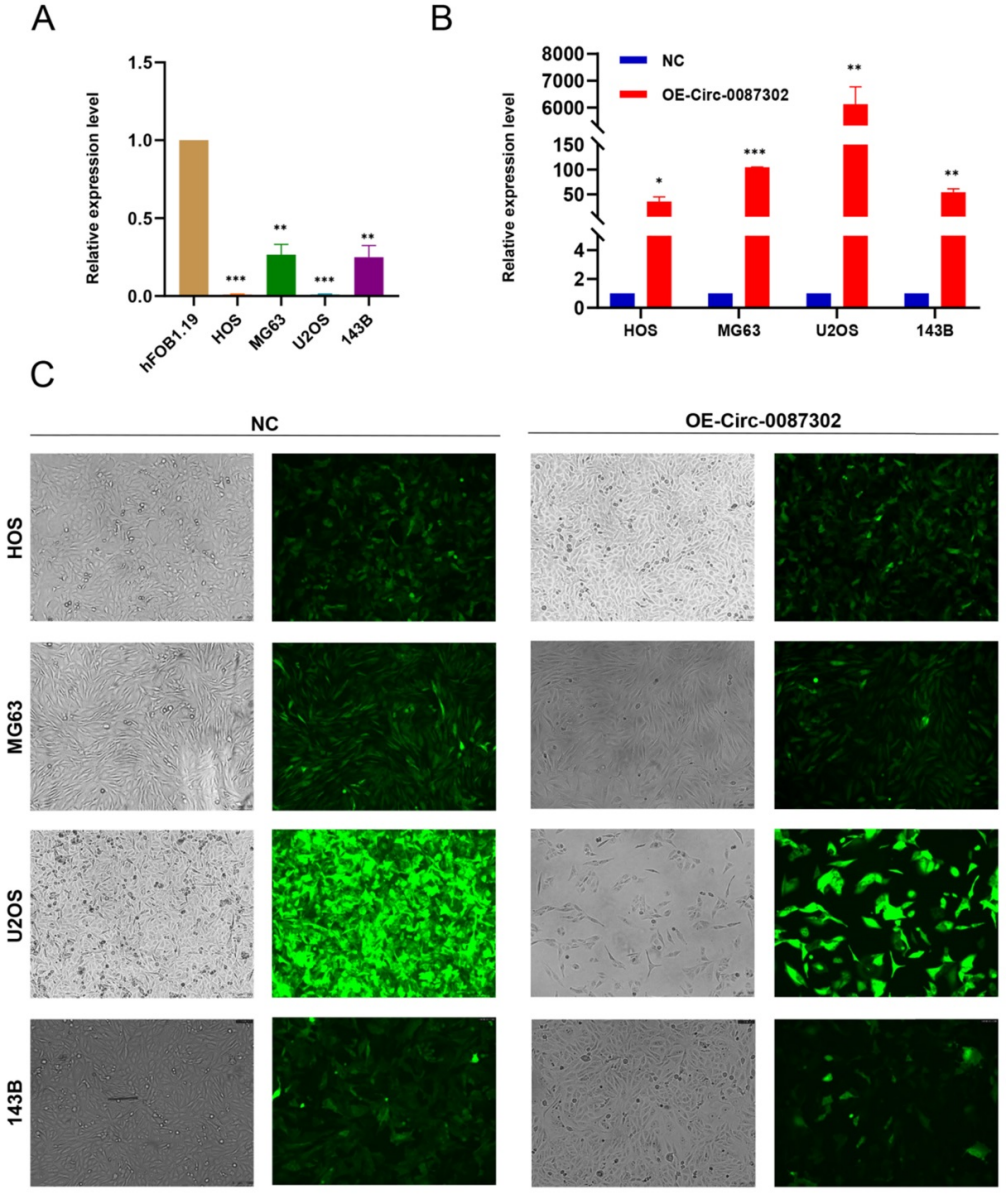
** The expression of hsa_circ_0087302 is decreased in osteosarcoma cells, and construction of the hsa_circ_0087302 overexpression model. (A)** The expression of hsa_circ_0087302 in OS cells was lower than that in osteoblasts. **(B-C)** The overexpression model was constructed by lentiviral transduction, and the transduction efficiency was verified by fluorescence microscopy and RT-PCR. NC is the negative control. Data represent the mean ± SD (n = 3) (*P < 0.05, **P < 0.01 and ***P < 0.001).

To study the effect of hsa_circ_0087302 on osteosarcoma, we first confirmed the reliability of sequencing results by verified the expression of hsa_circ_0087302 in four osteosarcoma cell lines and human osteoblasts by qRT-PCR. As expected, hsa_circ_0087302 was expressed at low levels in osteosarcoma cells (Figure 2A). To clarify the function of hsa_circ_0087302 *in vitro*, we constructed the hsa_circ_0087302 overexpression model by lentivirus transduction of osteosarcoma cells (HOS, MG63, U2OS and 143B). Subsequently, using puromycin to screen the successful transfected osteosarcoma cells. The bright green fluorescence of transfected osteosarcoma cells confirmed the successful transduction (Figure 2C). Additionally, qRT-PCR experiments was also employed to further quantify the transduction efficiency of osteosarcoma cells (Figure 2B). qRT-PCR results showed that the expression level of hsa_circ_0087302 in osteosarcoma cells after lentivirus transduction was significantly higher than that in control cells (Figure 2B). We selected MG63 and U2OS cells for subsequent analyses.

### Effects of hsa_circ_0087302 overexpression on cell proliferation and colony formation ability

**Figure 3:**
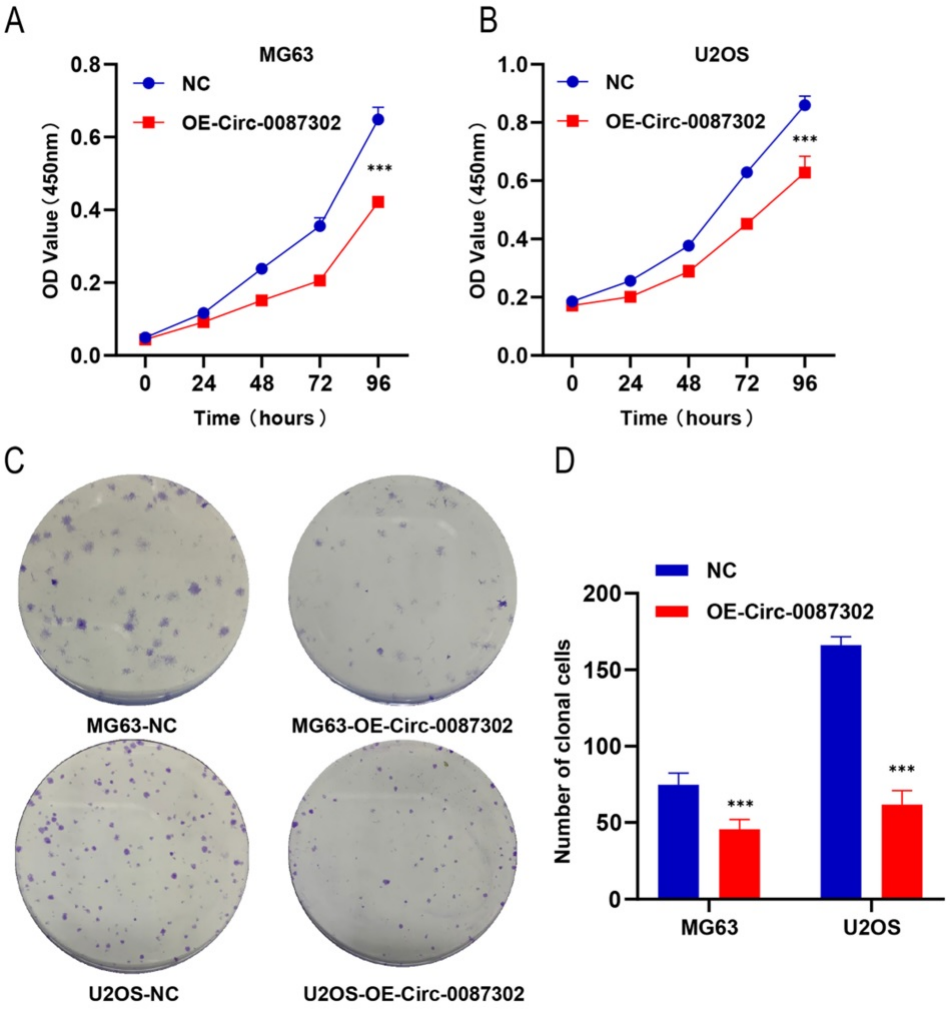
** Effect of hsa_circ_0087302 overexpression on the proliferation of osteosarcoma cells. (A-B)** A CCK-8 assay was used to detect cell viability. **(C-D)** A colony formation assay was used to detect cell colony formation. The bar chart shows the number of colonies with more than 50 cells. NC is the negative control. Data represent the mean ± SD (n = 3) (*P < 0.05, **P < 0.01 and ***P < 0.001).

To explore the effect of hsa_circ_0087302 on the proliferation and clonogenicity of osteosarcoma cells, the CCK-8 assay and colony formation assays were performed when the osteosarcoma cells were in the logarithmic growth phase. The CCK-8 assay results showed that the increased expression of hsa_circ_0087302 decreased the viability of osteosarcoma cells compared with that of control cells after four days of culture (Figure 3A-B) (P value < 0.05). The colony formation assay results were consistent with those of the CCK-8 assay. The number of colony formation decreased with the increase of hsa_circ_0087302 expression level compared with that of control cells (Figure 3C) (P value < 0.05). These results suggested that hsa_circ_0087302 may inhibit tumor growth.

### Effects of hsa_circ_0087302 overexpression on the osteosarcoma cell cycle

**Figure 4:**
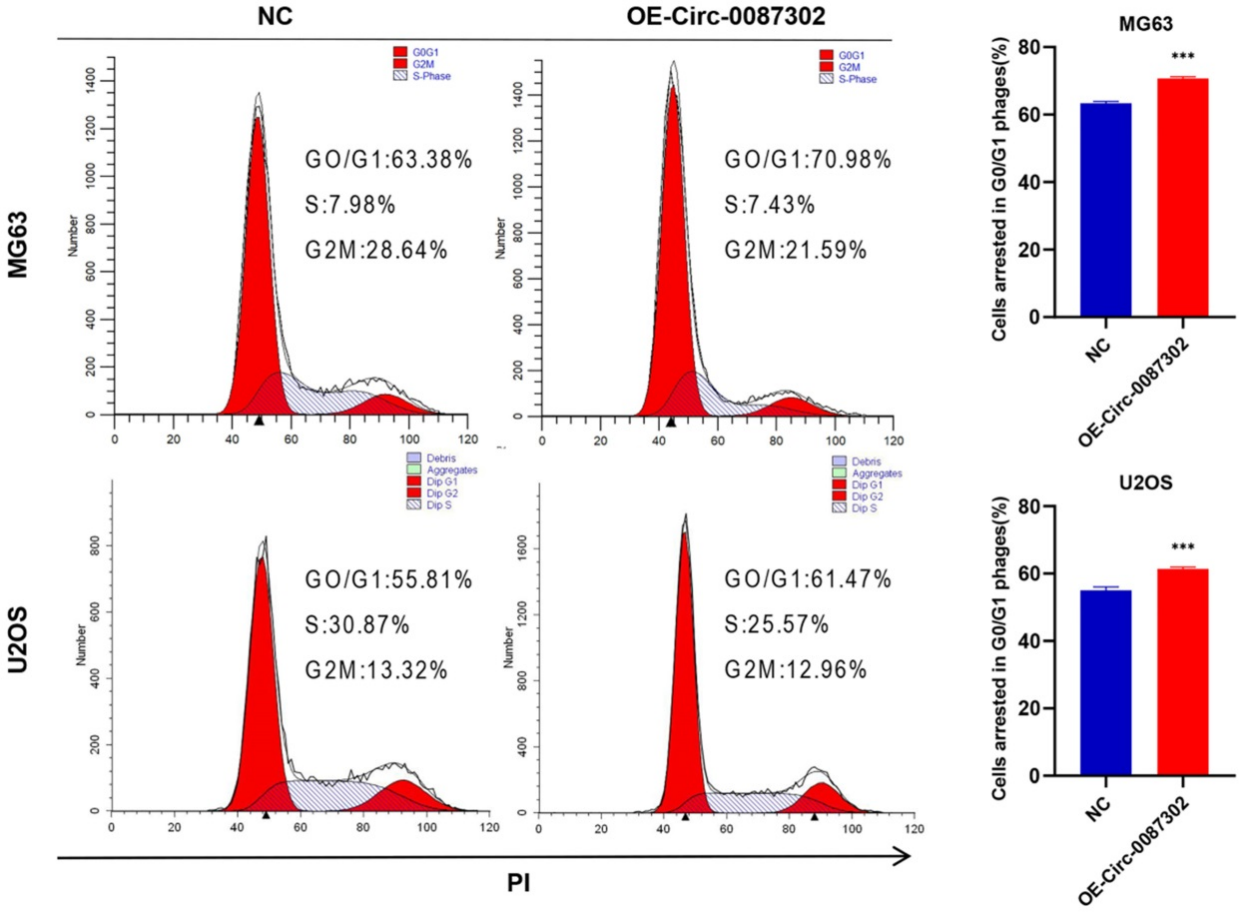
** Effect of hsa_circ_0087302 on the osteosarcoma cell cycle.** Changes in the cell cycle of osteosarcoma cells transduced with lentivirus were detected by flow cytometry. The bar chart shows the change in the G0/G1 phase. NC is the negative control. Data represent the mean ± SD (n = 3) (*P < 0.05, **P < 0.01 and ***P < 0.001).

Because decreased proliferation may be caused by an aberrant cell cycle, flow cytometry was used to analyze the influence of hsa_circ_0087302 on the OS cell cycle. Flow cytometry analysis indicated that hsa_circ_0087302 overexpression led to G0/G1 phase accumulation (Figure 4). These findings suggested that hsa_circ_0087302 may participate in the regulation of the cell cycle in osteosarcoma cells.

### Effects of hsa_circ_0087302 overexpression on osteosarcoma cell migration and invasion

**Figure 5:**
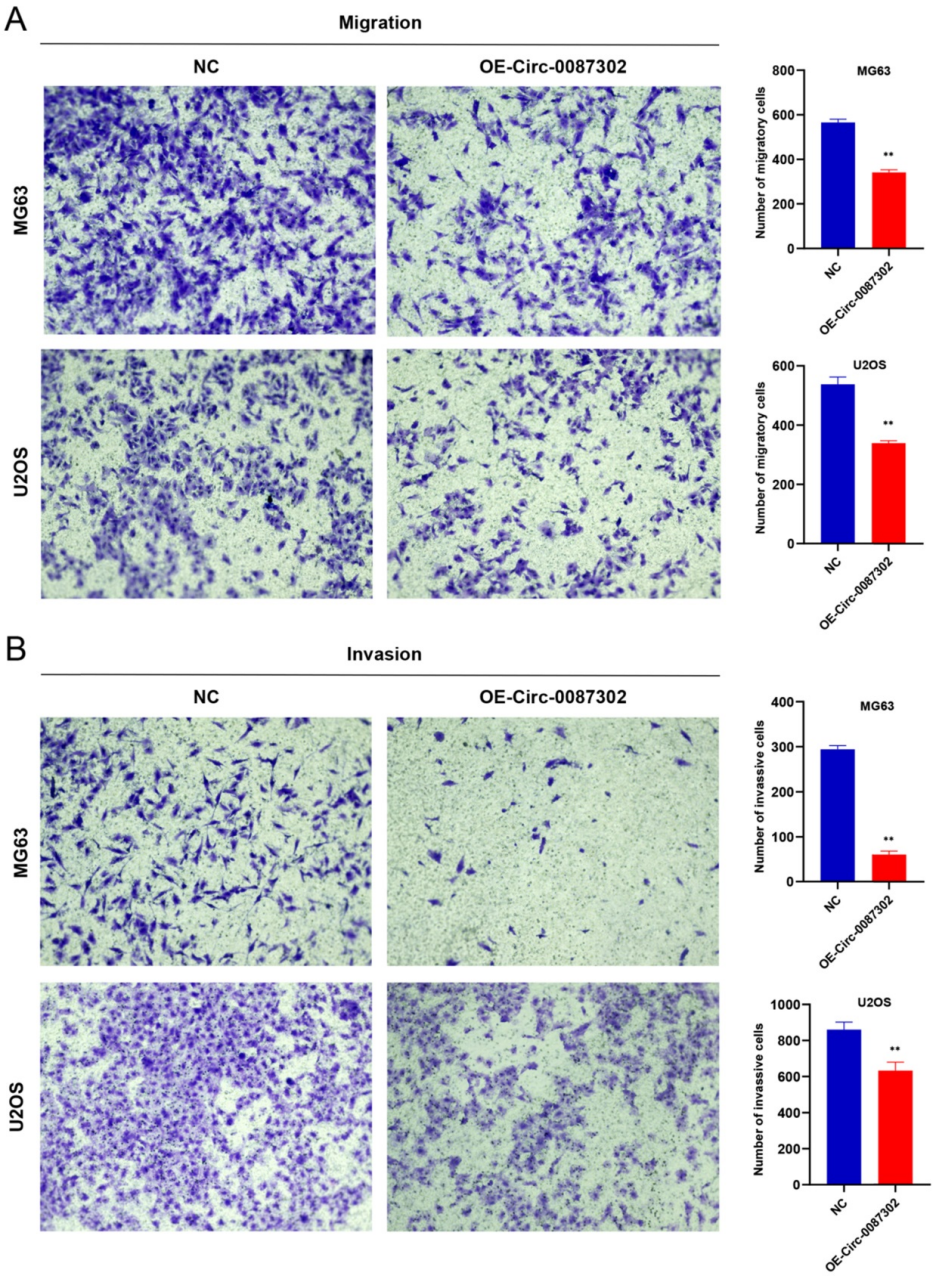
Overexpression of hsa_circ_0087302 reduced cell migration and invasion. Lentiviral transduction in MG63 and U2OS cells reduced cell migration (**A**) and invasion (**B**). All images were acquired using 10x magnification. The bar chart shows the number of cells passing through the surface of the Transwell. NC is the negative control. Data represent the mean ± SD (n = 3) (*P < 0.05, **P < 0.01, ***P < 0.001).

The high metastasis rate of osteosarcoma patients may be closely related to cell migration and invasion. To verify the effect of hsa_circ_0087302 on the migration and invasion of osteosarcoma cells, we performed Transwell assays. Overexpression of hsa_circ_0087302 decreased the number of migratory and invasive cells compared with the controls (Figure 5A-B) (P value < 0.05), indicating that hsa_circ_0087302 may be involved in the mechanism of tumor metastasis.

### Hsa_circ_0087302 affects the progression of osteosarcoma through the Wnt/β-catenin signaling pathway

**Figure 6:**
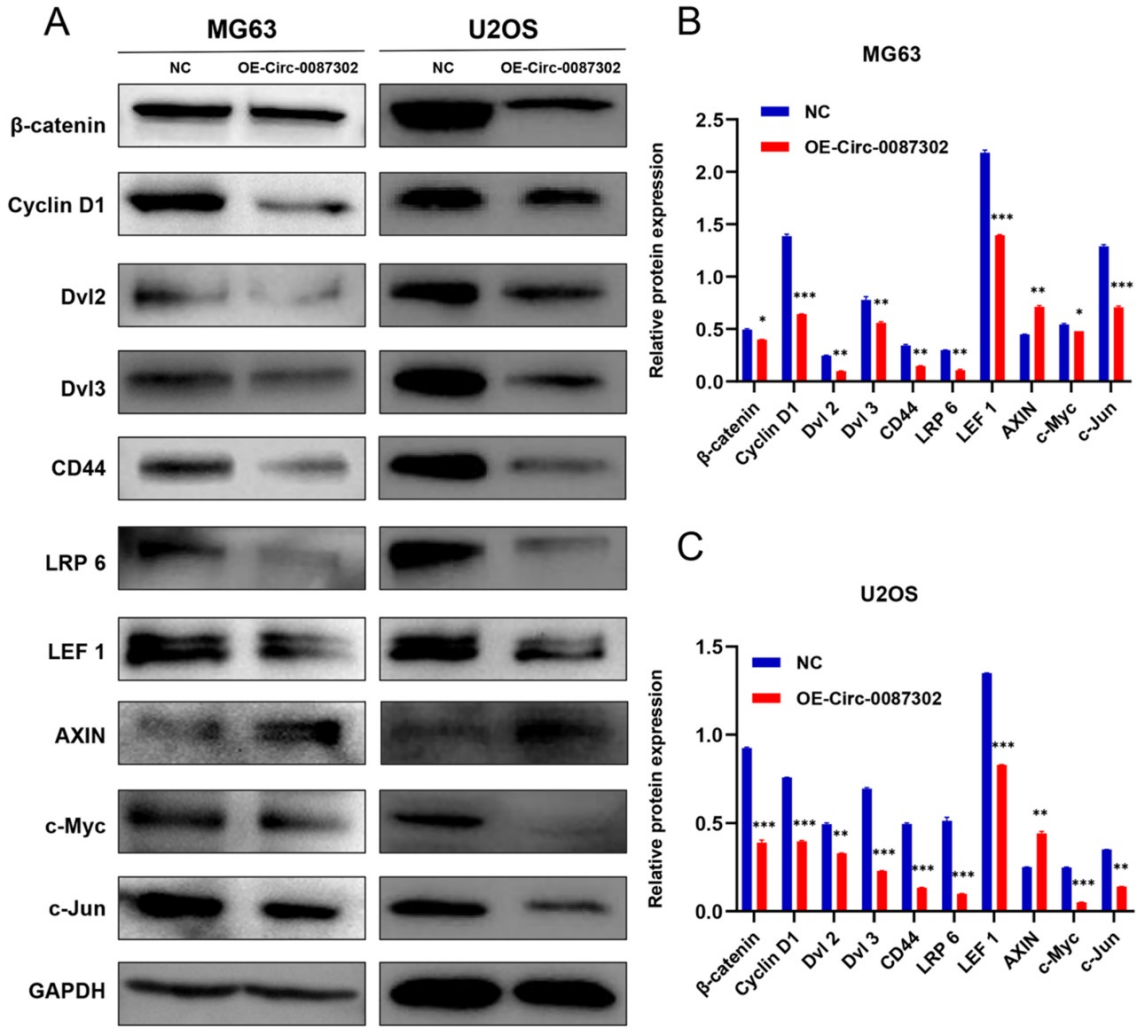
** Expression level of Wnt/β-catenin signaling pathway-related proteins after overexpression of hsa_circ_0087302.** (**A**) The protein levels of β-catenin, cyclin D1, Dvl 2, Dvl 3, CD44, LRP 6, LEF 1, c-Myc and c-Jun were downregulated by lentiviral transduction in MG63 and U2OS cells, while the protein level of Axin was increased. GAPDH was used as a loading control. The bar chart shows the expression level of each protein in MG63 (**B**) and U2OS (**C**) cells compared to control cells. NC: negative control. Data represent the mean ± SD (n = 3) (*P < 0.05, **P < 0.01 and ***P < 0.001).

To explore the molecular mechanism of hsa_circ_0087302 on the proliferation, migration and invasion of osteosarcoma cells, according to the results of KEGG database, we detected the key node proteins in Wnt/β-catenin signaling pathway which was closely related to lysine degradation, the results were as expected. We found that the protein levels of β-catenin, cyclin D1, Dvl 2, Dvl 3, CD44, LRP 6, LEF 1, c-Myc and c-Jun decreased but that the protein level of Axin increased with the increase of hsa_circ_0087302 expression (Figure 6A-B). These findings suggested that hsa_circ_0087302 may participate in the biological behavior of osteosarcoma via the Wnt/β-catenin signaling pathway.

## Discussion

In the present study, we identified differentially expressed circRNAs in osteosarcoma cells and normal human osteoblasts through RNA sequencing. Because the relationship between most differentially expressed circRNAs and osteosarcoma is unclear, we studied the specific mechanism between circRNA and osteosarcoma by focusing on hsa_circ_0087302. Compared with human osteoblasts, we verified the low expression of hsa_circ_0087302 in osteosarcoma cells. Hsa_circ_0087302 overexpression inhibited the rapid proliferation of osteosarcoma cells by affecting cell proliferation, colony formation, and at the same time causes changes in the cell cycle. Studies have shown that cell cycle dysregulation is an important factor in the abnormal proliferation of tumor cell 30. TP53 is a transcription factor that stabilizes and induces cell cycle arrest after genotoxic stress to function as a tumor suppressor, however, mutated TP53 not only causes cell cycle dysregulation, but also enhances the proliferative capacity of osteosarcoma cells, the study found that in a pig model, the mutated TP53 and its circular counterpart play an important role in the occurrence and progression of osteosarcoma and various cancers 31. In addition, overexpression of hsa_circ_0087302 inhibited cell migration and invasion. Studies have shown that the high metastasis rate of osteosarcoma is closely related to the enhancement of cell migration and invasion ability 32, which indicates that the abnormal expression of hsa_circ_0087302 is involved in the biological behavior of osteosarcoma metastasis.

The occurrence and development of osteosarcoma involves a variety of complex molecular mechanisms and different signal transduction pathways 33,34. KEGG pathway enrichment analysis showed that the differentially expressed circRNAs were closely related to lysine degradation. Lysine degradation mainly through four pathways, including pipecolic acid pathway, saccharopine pathway, acetyllysine pathway and the lysine-urea cycle 35. The relationship between lysine acetylation and Wnt/β-catenin signaling pathway is of great significance to the occurrence and progression of tumors. Histone lysine acetylation can regulate the transcription of important biomolecules in the Wnt/β-catenin signaling pathway, while non-histones Lysine acetylation directly alters the function of core molecules in Wnt/β-catenin signaling pathway 36. Previous studies have shown that the Wnt/β-catenin signaling pathway plays an important role in the occurrence and progression of osteosarcoma 37,39. Therefore, we detected the expression of several key proteins in the Wnt/β-catenin signaling pathway, including the upstream regulators (LRP 6, Dvl 2 and Dvl 3) and downstream targets (CD44, cyclin D1, LEF 1, c-Myc and c-Jun) of β-catenin. In addition, we also detected Axin which associated with β-catenin degradation. Hsa_circ_0087302 overexpression decreased the expression levels of key proteins, except Axin, in osteosarcoma cells. These findings suggested that tumor cells overexpressing hsa_circ_0087302 inhibit tumor progression by inhibiting the activation of the Wnt/β-catenin signaling pathway. As a single-pass transmembrane receptor, LRP6 interacts with the frizzled (FZD) family and activates the Wnt/β-catenin signaling pathway, which has been demonstrated in many cancers, including breast cancer, prostate cancer, hepatocellular carcinoma, and retinoblastoma 40,42. Dvl2 and Dvl3 are important mediators of the Wnt/β-catenin signaling pathway signaling transduction pathway. Studies have shown that DVL overexpression is important for the activation of the Wnt/β-catenin signaling pathway and cell growth in tumor cells 43,44. The decrease of LRP6, Dvl2 and Dvl3 expression inhibited Wnt signal transduction. Axin is involved in the degradation of β-catenin and negatively regulates the Wnt/β-catenin signaling pathway signal transduction pathway and inhibits the proliferation, invasion and tumorigenicity of osteosarcoma 45. The increase of axin expression leads to the increased degradation of β-catenin and then inhibits the transduction of Wnt/β-catenin signaling pathway. LEF1 is a member of the high mobility group (HMG) DNA-binding protein family of transcription factors, which mainly regulates the transcription of downstream target genes by β-catenin 46. As a type I transmembrane glycoprotein, CD44 participates in the migration and invasion of osteosarcoma 47. CyclinD1, as a regulator of the cell cycle, affects the proliferation of tumor cells through cell cycle arrest 48. As an oncogene, c-Myc has been found to be involved in many biological behaviors of tumor cells, such as cell proliferation, apoptosis and the cell cycle 49. Moreover, as the downstream target gene of the ErbB signaling pathway and central carbon metabolism in cancer, c-Myc participates in the malignant biological behavior of tumors, which is also in accordance with the KEGG pathway enrichment analysis. C-Jun has been reported to be involved in the biological behavior of migration and invasion of osteosarcoma 50. Here, we found that hsa_circ_0087302 overexpression may reduce Wnt signal transduction by down regulating the expression of LRP6, Dvl2 and Dvl3, increase the degradation of β-catenin and inhibit the transmission of Wnt signal by causing the upregulation of Axin expression. The downregulation of β-catenin content and abnormal Wnt signal transduction leads to the decrease of β-catenin entry into the nucleus, resulting in the decrease of the expression of transcription factor LEF1 and downstream target gene CD44, cyclin D1, c-Myc and c-Jun by β-catenin. Thus hsa_circ_0087302 mainly affects the progression of osteosarcoma via the Wnt/β-catenin signaling pathway. However, the downstream target and specific binding protein of hsa_circ_0087302 remain unclear, and the specific mechanism of the Wnt/β-catenin signaling pathway in osteosarcoma needs to be further clarified.

In summary, the present study showed that hsa_circ_0087302 may be directly involved in the malignant biological behavior of osteosarcoma, such as proliferation, cell cycle, migration and invasion, through the Wnt/β-catenin signaling pathway. In the near future, hsa_circ_0087302 may become a specific therapeutic target for osteosarcoma, effectively improving the comprehensive diagnosis and treatment of osteosarcoma patients.
